# Bio-samtools: Ruby bindings for SAMtools, a library for accessing BAM files containing high-throughput sequence alignments

**DOI:** 10.1186/1751-0473-7-6

**Published:** 2012-05-28

**Authors:** Ricardo H Ramirez-Gonzalez, Raoul Bonnal, Mario Caccamo, Daniel MacLean

**Affiliations:** 1 The Genome Analysis Centre, Norwich Research Park, Colney Lane, Norwich, NR4 7UH, UK; 2 Istituto Nazionale Genetica Molecolare, Via F. Sforza 28, Milan 20122, Italy; 3 The Sainsbury Laboratory, Norwich Research Park, Colney Lane, Norwich, NR4 7UH, UK

**Keywords:** Next-generation sequencing, DNA, High, Throughput, Ruby, Bio, SAM, BAM

## Abstract

**Background:**

The SAMtools utilities comprise a very useful and widely used suite of software for manipulating files and alignments in the SAM and BAM format, used in a wide range of genetic analyses. The SAMtools utilities are implemented in C and provide an API for programmatic access, to help make this functionality available to programmers wishing to develop in the high level Ruby language we have developed bio-samtools, a Ruby binding to the SAMtools library.

**Results:**

The utility of SAMtools is encapsulated in 3 main classes, Bio::DB::Sam, representing the alignment files and providing access to the data in them, Bio::DB::Alignment, representing the individual read alignments inside the files and Bio::DB::Pileup, representing the summarised nucleotides of reads over a single point in the nucleotide sequence to which the reads are aligned.

**Conclusions:**

Bio-samtools is a flexible and easy to use interface that programmers of many levels of experience can use to access information in the popular and common SAM/BAM format.

## Background

High-throughput DNA sequencing in the biological sciences has made it possible for researchers to obtain many millions of sequence reads in single, low-cost experiments. These sequence reads are typically very short compared to the parent genome (reads will usually be in the range of 36 - 200 nucleotides long while genomes are many millions of nucleotides long) and very redundant; many reads may have the same sequence. One widespread use for these sequences is in detecting small differences in the genome sequence of the sample donor, which is achieved by using computational methods to align each short sequence read against a long, reference genome sequence then examining the derived alignments and determining positions at which there are differences. Many programs have been created for alignment including BWA [1], Bowtie [2], SOAP [3], NOVOALIGN [4] and BFAST [5], each implementing different algorithms optimised to address different issues with the alignment problem. Most high-throughput alignment programs produce a standard output file in Sequence Alignment/Map format (SAM) [6], a tab-delimited text-based format for describing alignments. The SAMtools utilities comprise a very useful and widely used suite of software for manipulating files and alignments in the SAM format. The large SAM files can be converted to the binary equivalent BAM files a compressed and indexed variant for random access, which vastly facilitates genetic analyses that rely on high-throughput alignment. The SAMtools utilities are implemented in C and provide an API for programmatic access, for which there are multiple language bindings, notably in Perl [7], Python [8] and Java [9]. Here we describe the Ruby language binding to the SAMtools library, developed for our own work and distributed as a BioRuby plug-in [10].

## Implementation

**Figure 1:**
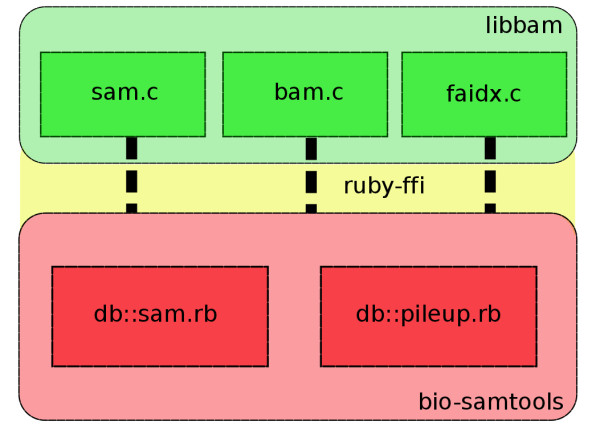
**bio-samtools and its relationship to the underlying libbam.** Green boxes indicate C source code in libbam, red boxes indicate Ruby files that interact the Ruby FFI represented in yellow.

The bio-samtools package is a wrapper around libbam.so (for Linux) and libbam.1.dylib (for Mac OS X), the core shared object library from the SAMtools package. To make it possible for the C functions in libbam to be called from within Ruby code we have used the Ruby Foreign Function Interface (FFI) [11] package as a bridge between the two languages. The flexible FFI package can programatically load dynamic libraries and bind functions without the need to make changes to Ruby itself or to recompile any extensions, so our package can easily run on standard Ruby interpreters without installation and compilation issues beyond that of the normal Ruby gem installation. Importantly, FFI also has useful methods for managing memory, pointers, structs and binary fields are converted to Ruby boolean variables. A further advantage of using FFI is that the binding is compatible with both the standard Ruby interpreter Matz’s Ruby Interpreter (MRI) and the alternative Java implementation of the Ruby language (JRUBY). By wrapping SAMtools in this way the scientist may use the high level easily learned and fast to develop with Ruby that facilitates quick development rather than the native C of SAMtools. bio-samtools hides the low-level API completely making bio-samtools a useful and easily used tool for working with Next-Generation Sequencing data in BAM files. Each .c library from the SAMtools API is represented by a separate Ruby module mapping the C functions (Figure 1), which are unified in the class Sam.

### Bio::DB::Sam

The main object representing the SAM/BAM file is a Bio::DB::Sam object. Objects of class Sam, have several attributes and methods, summarised in Table 1. Most of the attributes relate to the alignment file type and the location of the BAM file in the file system. The BAM file itself is not held in the object or Ruby memory, rather the Ruby wrapping is used to access the information via the C API. The methods of the Sam object can be divided into utility methods that affect the BAM files, (#sort and #merge), retrieval methods that return objects of other classes representing individual read alignments (#fetch, #fetch_with_function) and summary methods (#average_coverage, #chromosome_coverage and #mpileup,#index_stats).

**Table 1:** Attributes and methods of the Bio::DB::Sam object

binary	denotes whether this is a binary file
compressed	denotes whether this file is compressed
fasta_path	path to the reference FASTA file
sam	path to the associated BAM file
chromosome_coverage	return Ruby Array of coverage over a region
fetch	fetch alignment in a region from a bam file, returning a Ruby Array object
fetch_reference	fetch regions of the reference file returning a String object of the relevant sequence
fetch_with_function	fetch all alignments in a region passing in a Ruby Proc object as a callback, returning an iterator
index_stats	get information about reference and number of mapped reads
merge	merge two or more bam files
mpileup	an iterator that returns Pileup objects representing the reads over a single position
sort	sort the BAM file

### Bio::DB::Alignment

The fetch and fetch_with_function method of the Bio::DB::Sam object return individual alignments one at a time from an iterator. The individual alignments represent a single read and its mapping to the reference and are Bio::DB::Alignment objects, whose attributes are described in Table 2. These attributes are derived directly from the SAM format definition [6]. The fetch_with_function method is distinct from fetch in that it allows the user to pass a Ruby Proc object or a block. These are functionally equivalent to closures in other languages and provide advantages in terms of encapsulation and often speed compared to the standard block based equivalent, advanced Ruby programmers are likely to appreciate this feature.

**Table 2:** Attributes of the Bio::DB::Alignment object

calend	nucleotide position of the end of the alignment
cigar	CIGAR string describing the matches/mismatches
failed_quality	this read failed the quality threshold
first_in_pair	first of a pair
is_duplicate	this read is a suspected optical or PCR duplicate
is_mapped	the read was aligned
is_paired	the read is one of a pair
isize	the insert size distance between mapped mates
mapq	the PHRED scaled mapping quality of the alignment
mate_strand	the strand of the mate
mate_unmapped	the mate is unmapped
mpos	start position of the mate on the reference
pos	start position of the alignments
primary	is a primary alignment
qlen	read length
qname	read name
qual	read quality string
query_strand	strand of alignment
query_unmapped	query is unmapped
rname	name of reference to which read mapped
second_in_pair	this is second in the pair
seq	read sequence
tags	Bio::DB::Tag object representing the tags for this alignment

### Bio::DB::Pileup

The pileup format is a straightforward way of structuring alignments over single positions for the easy identification of genetic polymorphisms, the format has a long history and has been in use in SAMtools for a while. The original ’pileup’ function has recently been deprecated and removed in favour of ’mpileup’. The output from mpileup is exactly equivalent to the pileup command called without the -c flag set, that is to say the six column format. The class Pileup can parse the old ten column pileup format if an instance is created manually by passing it a raw line from the pileup file. Calling the mpileup method of a SAM object results in the return of a stream of Pileup [12] objects, which have the attributes and methods listed in Table 3. Some of the attributes are related to the ten column format only. Notably, SAMtools will calculate a consensus base call if asked to return a ten column pileup file, so the Pileup class will use SAMtools consensus call if it is available, otherwise it will call a consensus based on a simple majority count.

**Table 3:** Attributes and methods of the Bio::DB::Pileup object

consensus	the consensus nucleotide calculated as the nucleotide with highest count multiple nucleotides returned in a tie
coverage	the number of reads covering this position
non_ref_count	the number of reads that disagree with the reference nucleotide
non_ref_count_hash	a Hash with A,T,G and C as keys and the number each nucleotide appears in the pileup when that nucleotide is not
	the reference
pos	the position in the reference sequence that this pileup represents
read_bases	the read nucleotides covering this position
read_quals	the quality scores of the read nucleotides covering this position
ref_base	the reference sequence nucleotide
ref_count	the number of times the reference nucleotide appears in the read nucleotides covering this position
ref_name	the name of the reference sequence
ar1, ar2, ar3	the allele calls from pileup
consensus^1^	the consensus of the reads according to SAMtools method of calculation
consensus_quality^1^	the quality score of the consensus according to SAMtools method of calculation
rms_mapq^1^	the root mean square mapping quality at the position
snp_quality^1^	the SNP quality at the position

^1^ten column format only.

## Results and discussion

### Using bio-samtools: a brief tutorial

bio-samtools in use is straightforward, here are a few examples of interacting with BAM files with the package. More information on specific functions is provided in the RubyDoc documentation and in the files bioruby-samtools/doc/tutorial.html and bioruby-samtools/doc/tutorial.pdf. The location of the bio-samtools installation folder can be found by typing ’gem which bio-samtools’ at the command-line.

#### Installation

bio-samtools is easily installed from a machine with an internet connection and a Ruby installation with the straightforward Gem invocation ’gem install bio-samtools’. bio-samtools automatically downloads the original libbam C source code and compiles it for Linux or OSX as appropriate. The new version of the library is kept locally to the bio-samtools code to avoid conflicts with other installations of the library.

#### Loading a BAM file

A SAM object represents the alignments in the BAM file, and is very straightforward to create, you will need a sorted BAM file, to access the alignments and a reference sequence in FASTA format to use the reference sequence. The object can be created and opened as follows:require 'bio-samtools'bam=Bio::DB::Sam.new(:bam=>"my_sorted.bam", :fasta=>'ref.fasta')bam.openbam.close

Opening the file needs only to be done once for multiple operations on it, access to the alignments is random so you don’t need to loop over all the entries in the file, as you would with a manual SAM file parse.

#### Getting summary information

The length of reference sequences and the number of reads mapped to each can be obtained with the index_stats function. A Hash object, keyed by reference name and with a Hash at each value is returned. The Hash at the value has keys :length, :mapped_reads and :unmapped_reads and values for each of these. The index_stats function wraps the SAMtools idxstats command.sam.index_stats# returns { "chr_1"=> {:length=>69930,:mapped_reads=>1000,:unmapped_reads=>0 }, }

#### Retrieving reference sequence

Retrieving the reference can only be done if the reference has been loaded, which isn’t done automatically in order to save memory. Reference need only be loaded once, and is accessed using reference name, start, end in 1-based co-ordinates. A standard Ruby String object is returned. In this example a 500 nucleotide region from the start of the sequence is returned.bam.load_referenceseq = bam.fetch_reference("Chr1", 1, 500)

#### Retrieving alignments in a region

Alignments in a region of interest can be obtained one at a time by giving the region to the fetch() function.bam.fetch("Chr1", 3000, 4000).each do | alignment |puts alignment.qname #do something with the alignment objectend

#### Get a summary of coverage in a region

It is easy to get the total depth of reads at a given position, the chromosome_coverage function is used. This differs from the previous functions in that a start position and length (rather than end position) are passed to the function. An array of coverages is returned, eg [26,26,27 .. ]. The first position in the array gives the depth of coverage at the given start position in the genome, the last position in the array gives the depth of coverage at the given start position plus the length given.coverages = bam.chromosome_coverage("Chr1", 3000, 1000)Similarly, average (arithmetic mean) of coverage can be retrieved, also with start and length parametersav_cov = bam.average_coverage("Chr1", 3000, 1000)

#### Getting pileup information

Pileup format represents the coverage of reads over a single base in the reference. Getting a Pileup over a region is very easy. Note that this is done with mpileup and NOT the now deprecated and removed from SAMTools pileup function. Calling the mpileup method creates an iterator that yields a Pileup object for each base.bam.mpileup do |pileup|puts pileup.consensusend

The mpileup function takes a range of parameters to allow SAMTools level filtering of reads and alignments. They are specified as key, value pairs. In this example a region is specified by :r and a minimum per base quality score is specified by :Q.bam.mpileup(:r => "Chr1:1000-2000", :Q => 50) do |pileup|puts pileup.coverageend

Not all the options SAMTools allows you to pass to mpileup are supported, those that cause mpileup to return Binary Variant Call Format (BCF) [13] are ignored. Specifically these are g,u,e,h,I,L,o,p. Table 4 lists the SAMTools flags supported and the symbols you can use to call them in the mpileup command.

**Table 4:** SAMtools options recognised by the Bio::DB:Sam#mpileup method and the symbols used to invoke them

**SAMTools option**	**description**	**short symbol**	**long symbol**	**default**
r	limit retrieval to a region	:r	:region	all positions
6	assume Illumina scaled quality scores	:six	:illumina_quals	FALSE
A	count anomalous read pairs scores	:A	:count_anomalous	FALSE
B	disable BAQ computation	:B	:no_baq	FALSE
C	parameter for adjusting mapQ	:C	:adjust_mapq	0
d	max per-BAM depth to avoid excessive memory usage	:d	:max_per_bam_depth	250
E	extended BAQ for higher sensitivity but lower specificity	:E	:extended_baq	FALSE
G	exclude read groups listed in FILE	:G	:exclude_reads_file	FALSE
l	list of positions (chr pos) or regions (BED)	:l	:list_of_positions	FALSE
M	cap mapping quality at value	:M	:mapping_quality_cap	60
R	ignore RG tags	:R	:ignore_rg	FALSE
q	skip alignments with mapping quality smaller than value	:q	:min_mapping_quality	0
Q	skip bases with base quality smaller than value	:Q	:imin_base_quality	13

## Conclusions

Ruby is an easily written and understood high-level language, ideal for beginners or those wishing to develop analysis scripts and prototype applications in short timeframes. A major advantage of scripting in Ruby for biologists is the BioRuby project that provides a lot of classes and functionality for dealing with common biological data types and file formats. bio-samtools is a BioRuby plugin which extends the original BioRuby framework by providing a useful and flexible interface for Ruby coders who wish to have programmatical access to the data in BAM and SAM files without losing performance, the C API is very much quicker than a pure Ruby implementation would be and wrapping it provides the best of both languages. The interface we provide gives access to all the API components of the SAMtools core library libbam.so and extends with some useful high level methods. The open class system of Ruby means that the SAM class which encapsulates the functionality of SAMtools can easily be extended at run-time by the user. These features together mean that bio-samtools can be an extremely useful tool for scientists wishing to examine the results of next-generation sequencing alignments.

## Availability and requirements

**Project name:** bio-samtools **Project home page:**http://rubygems.org/gems/bio-samtools**Operating systems:** Linux and Mac OS X **Programming language:** Ruby **Other requirements:** none **License:** as BioRuby **Any restrictions to use by non-academics:** none

## Competing interests

The authors declare that they have no competing interests.

## Authors’ contributions

RRG wrote the binding, tests, co-wrote the documentation and co-wrote the manuscript, RB created and organised the Gem and contributed to the binding and tests and co-wrote the manuscript and DM conceived of the binding, contributed to the binding and tests, tested the implementations with sample data and co-wrote the manuscript. RRG and RB contributed equally to this work. All authors read and approved the final manuscript.
